# Electrocatalytic Oxidation of HMF to FDCA over Multivalent Ruthenium in Neutral Electrolyte

**DOI:** 10.3390/molecules30081780

**Published:** 2025-04-15

**Authors:** Shiying Yang, Xin Jin, Bin Zhu, Dan Yang, Xiaoyue Wan, Yihu Dai, Chunmei Zhou, Yuguang Jin, Yanhui Yang

**Affiliations:** 1 Institute of Advanced Synthesis, School of Chemistry and Molecular Engineering, Jiangsu National Synergetic Innovation Centre for Advanced Materials, Nanjing Tech University, Nanjing 211816, China; yangshiying0824@163.com (S.Y.); jx202261205159@njtech.edu.cn (X.J.); yangdan@njtech.edu.cn (D.Y.); ias_xywan@njtech.edu.cn (X.W.); ias_yhdai@njtech.edu.cn (Y.D.); yhyang@njtech.edu.cn (Y.Y.); 2 Laboratory of Polymers and Composites, Ningbo Institute of Materials Technology and Engineering, Chinese Academy of Sciences, Ningbo 315201, China; 3 State Key Laboratory for Oxo Synthesis and Selective Oxidation, Lanzhou Institute of Chemical Physics, Chinese Academy of Sciences, Lanzhou 730000, China; jinyuguan@163.com

**Keywords:** 5-hydroxymethylfurfural, 2,5-furandicarboxylic acid, biomass platform chemicals, electrocatalytic oxidation, ruthenium oxide

## Abstract

5-Hydroxymethylfurfural (HMF) serves as an important bridge connecting biomass resources with fossil fuels. Its downstream product, 2,5-furandicarboxylic acid (FDCA), is a renewable alternative to terephthalic acid (TPA) in the synthesis of various polymer materials. In this study, we successfully synthesized four ruthenium-based catalysts with varying valence states supported on carbon nanotubes (CNTs) and compared the performance of HMF electrooxidation. Among these, the Ru^+2.9^ catalyst demonstrated the highest activity for the electrochemical oxidation of HMF to FDCA in the neutral medium (0.1 M K_2_SO_4_). Notably, the FDCA yield reached 90.2% under an applied potential of 0.95 V (vs. Ag/AgCl) after 24 h. Mechanistic analysis revealed that the superior specific capacitance of the Ru^+2.9^ catalyst significantly facilitated the reaction process. This work represents a more cost-effective approach to avoid the need for excessive alkaline additives during catalyst preparation and the HMF oxidation process, and FDCA separated easily after cooling the reaction solution down.

## 1. Introduction

Biomass fuel, as a promising sustainable energy source, has the widest distribution and largest reserves in the world [1,2]. Further processing and refining of this energy can produce more high-value-added chemicals and liquid fuels, which are expected to replace fossil fuels as a new energy source supporting social development to solve a series of problems such as serious environmental pollution and energy shortage caused by the continuous consumption of fossil fuels in recent years. 5-Hydroxymethylfurfural (HMF), as an important platform molecule connecting biomass raw materials and downstream high-value products [3], is obtained from fructose, glucose, cellulose, etc. converted from biomass hydrolysis. The catalytic oxidation products of HMF include important applications such as 2,5-diformylfuran (DFF), 5-hydroxymethyl-2-furanic acid (HMFCA), 5-formyl-2-furanic acid, and 2,5-furandicarboxylic acid (FDCA) [4] due to the presence of C=O, C-O, -OH, and furan ring functional groups. Among them, FDCA is considered by the US Department of Energy to be one of the twelve chemicals with the highest value added obtained from biomass, with the potential to replace terephthalic acid in the production of polyethylene terephthalate (PET), which is of great significance in addressing issues such as fossil fuel shortages and pollution [5,6].

In the early days, FDCA was mostly achieved through traditional oxidation of homogeneous catalytic systems by HMF under high air pressure, and these methods have become relatively mature [7,8]. Later, researchers began to study heterogeneous catalytic systems, using precious metals such as Pt [9], Pd [10], Au [11], and Ru [12] and non-precious metals [13] such as Fe, Co [14], Mn [15], and Cu [16] under the conditions of air and oxygen as oxidants. New technologies for HMF catalytic conversion are constantly being attempted [17,18], and electrochemistry is receiving increasing attention due to its unique advantages [19,20]. Compared with traditional thermal catalysis, electrocatalysis is carried out at room temperature and pressure without adding oxidants, and the selectivity of the reaction can be adjusted by changing the applied voltage. More importantly, the electrical signals output during the electrocatalytic process can be used to monitor the reaction process online, facilitating our study of kinetics. As early as 1991, Grobowski et al. [21] used NiO(OH) as catalyst to obtain 71% yield of FDCA through a simple electrocatalytic process. In the past five years, more and more reports have been reported. David J. Chadderdon [22] demonstrated the synergistic effect of Au-Pd on the generation of FDCA by the electrooxidation of HMF and revealed the unique catalytic properties of Pd and Au on the competitive oxidation of alcohols and aldehyde groups in HMF; Bo You et al. [23,24] studied the highly efficient catalytic activity of nickel-based catalyst for the oxidation of HMF under alkaline conditions and realized the simple and effective strategy of H_2_ production and biomass upgrading. The Faraday efficiency can reach 100%; Stefan Barwe et al. [25,26] explored that HMF oxidation proceeds via the HMFCA intermediate rather than the DFF pathway over B-modified NF, and the catalyst inhibited the decomposition of HMF under alkaline conditions to reduce the loss. However, the addition of alkali will complicate the process and cause corrosion to the equipment, which violates the principle of green and environmental protection technology. On the other hand, very few reports have been made regarding acidic conditions. They mainly focus on the selectivity of DFF, while the yield of FDCA is relatively low. But in 2021, our research group achieved 72.1% FDCA yield in neutral electrolytes using RuO_2_/MnO_2_/CNT catalyst for the first time [27].

In this study, four ruthenium-based (Ru) catalysts with different valence states were prepared using the plasma reduction method, ethylene glycol reduction method, impregnation reduction method, and NaBH_4_ reduction method. These four Ru catalysts were employed for the electrocatalytic oxidation of HMF, and their performance was comprehensively compared. Among them, the Ru^+2.9^ catalyst exhibited high electrooxidation activity in the formation of FDCA. The FDCA yield reached 90.2% under a potential of 0.95 V (vs. Ag/AgCl) after 24 h by the Ru^+2.9^ catalyst.

## 2. Results and Discussion

### 2.1. Fabrication and Characterization of the Ru Catalysts

**Figure 1:**
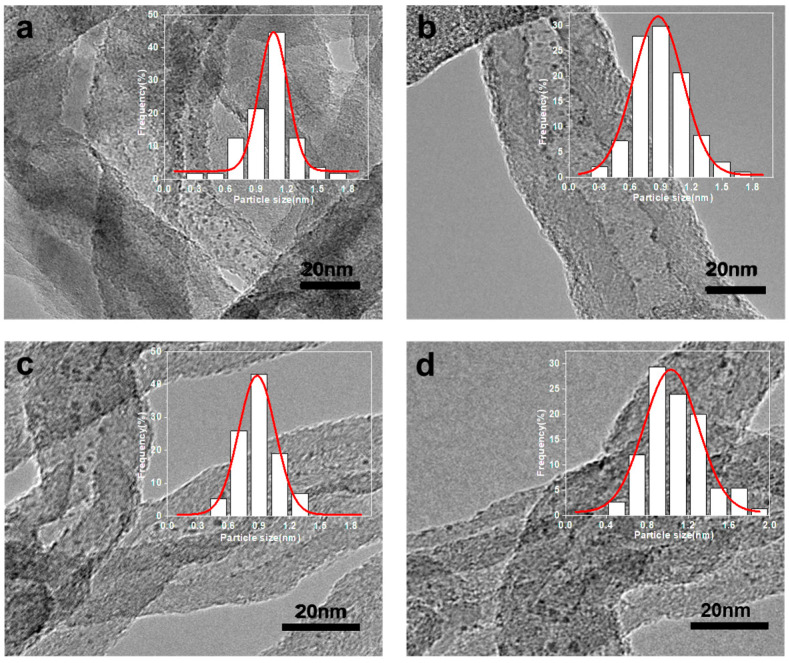
HR-TEM images and particle size distribution of (**a**) Ru-1, (**b**) Ru-2, (**c**) Ru-3, and (**d**) Ru-4 catalysts.

**Figure 2:**
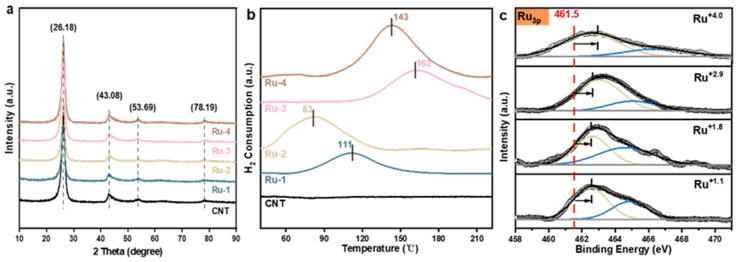
(**a**) XRD patterns of CNTs and four Ru catalysts. (**b**) The H_2_-TPR profiles of the four Ru catalysts and CNTs. (**c**) XPS spectra of Ru-1, Ru-2, Ru-3, and Ru-4 catalysts at Ru 3p orbit.

The microstructure and particle size distribution of Ru-based catalysts synthesized by four different methods were evaluated using high-resolution transmission electron microscopy (HR-TEM). As shown in Figure 1, the Ru particles in all four Ru-based catalysts are uniformly distributed on the carbon nanotubes (CNTs), with particle sizes of 1.02, 0.89, 0.92, and 1.08 nm, respectively. Figure 2a presents the X-ray diffraction (XRD) patterns of the CNTs and the Ru-based catalysts supported on CNTs. Almost the same diffraction peaks of 26.18°, 43.08°, 53.69°, and 78.19° can be attributed to the (002), (101), (004), and (110) planes supported by CNTs, indicating that the crystallinity among the four catalysts is similar and the Ru loading is relatively low. Inductively coupled plasma optical emission spectrometry (ICP-OES) measurements revealed that the Ru loadings of the four catalysts were 0.72, 0.68, 0.88, and 0.74 wt%, respectively.

The average oxidation state (AOS) of Ru in the catalyst was calculated using copper oxide as the internal standard through hydrogen adsorption (Figure 2b). The calculated Ru valence states for the Ru-1, Ru-2, Ru-3, and Ru-4 catalysts were +1.1, +1.8, +2.9, and +4.0, respectively. These results confirm the successful synthesis of ruthenium catalysts with different oxidation states in this study. Notable differences were observed in the reduction temperatures of the four catalysts, which followed the following trend: Ru^+2.9^ > Ru^+4.0^ > Ru^+1.1^ > Ru^+1.8^. Among them, the Ru^+2.9^ catalyst exhibited the highest reduction temperature (162 °C), attributed to the reduction of the Ru^+3^ precursor to metallic Ru^0^ [28]. The reduction temperatures of 143 °C, 111 °C, and 83 °C were observed for the in Ru^+4.0^, Ru^+1.1^, and Ru^+1.8^ catalysts, respectively, corresponding to the reduction of RuO_2_ (Ru^+4^) to Ru^0^ [29,30]. The different valence states of Ru alter the electronic structure of the catalyst, and the different valence states on the surface of Ru affect the catalytic activity of the reaction.

Further X-ray photoelectron spectroscopy (XPS) analysis of the catalysts was conducted, as shown in Figure 2c. The Ru 3d peak overlaps with the C 1s peak (284.6 eV), so the binding energy of the Ru 3p peak was used to evaluate the oxidation state of Ru on the catalyst surface. The spectra for the four catalysts were fitted into two peaks, designated as Peak 1 (red) and Peak 2 (blue), respectively. The binding energy of metallic Ru is 461.5 eV. For the carbon-loaded Ru catalyst synthesized in this study, the binding energies are higher than 461.5 eV, indicating that Ru is electron deficient for all carbon materials. Among the four catalysts, the Ru^+2.9^ catalyst exhibits the largest binding energy shift (1.5 eV), while the shifts for Ru^+4.0^, Ru^+1.1^, and Ru^+1.8^ are similar, at 1 eV, 1 eV, and 1.1 eV, respectively. The significant binding energy shift observed for Ru^+2.9^ suggests a stronger electron deficiency, which may facilitate the formation of active centers through interactions with -OH groups.

### 2.2. Electrooxidation of HMF over Ru Catalysts

**Figure 3:**
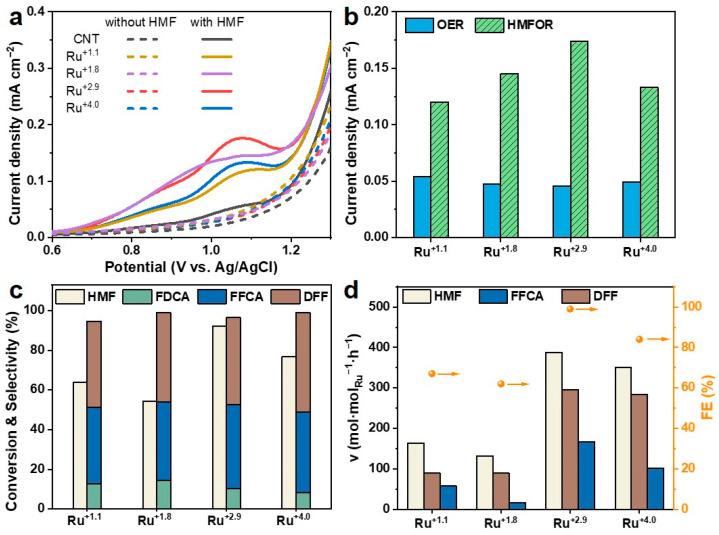
(**a**) LSV curves in 0.1 M K_2_SO_4_ with the absence and presence of HMF by the CNTs and four Ru catalysts. (**b**) The current density of OER and HMFOR by the four Ru catalysts at 1.1 V (vs. Ag/AgCl). (**c**) Conversion of HMF and yield of its oxidation products on the four Ru catalysts within 2 h. (**d**) The initial reaction rates for the conversion of intermediates and HMF on the four Ru catalysts within 30 min. Reaction conditions: HMF, 20 mM; K_2_SO_4_, 30 mL; temperature, 60 °C; voltage, 0.95 V (vs. Ag/AgCl).

The electrocatalytic performances of the catalysts were evaluated in a three-electrode system using a neutral 0.1 M K_2_SO_4_ aqueous electrolyte. The linear sweep voltammetry (LSV) curves are presented in Figure 3a to compare the catalytic activities of the CNTs and four Ru catalysts. In the absence of HMF, all five catalysts, including CNTs, exhibit negligible differences. Upon the addition of HMF, it is observed that the four Ru-based catalysts demonstrate a significant current density response compared to CNTs. This indicates that the electrocatalytic activity for HMF oxidation is derived from Ru. Further comparison among the four Ru catalysts reveals that the Ru^+2.9^ catalyst exhibits a stronger oxidation peak in the range of 1.0–1.1 V (vs. Ag/AgCl) compared to the other three catalysts. This suggests a superior biomass upgrading capability of the Ru^+2.9^ catalyst. As shown in Figure 3b, at a potential of 1.1 V (vs. Ag/AgCl), the current density of the Ru^+2.9^ catalyst increases from 0.046 mA cm^−2^ to 0.174 mA cm^−2^ upon the addition of HMF. In contrast, the current densities of Ru^+1.1^, Ru^+1.8^, and Ru^+4.0^ in the presence of HMF are 0.120, 0.145, and 0.133 mA cm^−2^, respectively. A higher current density can ensure the rapid conversion of HMF.

Figure 3c shows the conversion of HMF and selectivities of downstream products after electrolysis at 0.95 V (vs. Ag/AgCl) within 2 h. It was observed that, as the oxidation state of Ru increased, the catalytic performance exhibited a volcano-shaped trend. Among these, the Ru^+2.9^ catalyst demonstrated the best HMF conversion and DFF selectivity, reaching 92.3% and 44.1%, respectively. This is consistent with the results of LSV curves.

To further compare the catalytic abilities of the four catalysts, the initial reaction rates of the catalysts are presented in K_2_SO_4_ solutions with HMF, DFF, and 5-formylfuran-2-carboxylic acid (FFCA) as reaction substrates. The conversions of each substrate were controlled within 25%, and the initial reaction rates for each step on the four catalysts were calculated. The results are shown in Figure 3d. The initial reaction rate of each reactant in Ru^+2.9^ is significantly higher than that of other catalysts. The initial reaction rates of HMF, DFF, and FFCA are 388, 296, and 167 mol∙mol_Ru_^−1^∙h^−1^, respectively. HMF has the highest conversion rate, about 1.5 times that of Ru^+4.0^, 2.5 times that of Ru^+1.1^, and 3 times that of Ru^+1^. Additionally, Ru^+2.9^ achieved a Faradaic efficiency (FE) of 99% within 30 min, which was significantly higher than that of the other three catalysts. The highest Faraday efficiency confirms its stronger hydrogen electron transfer ability. For the reaction rate of DFF, the order is Ru^+2.9^ > Ru^+4.0^ > Ru^+1.1^ ≥ Ru^+1.8^. Specifically, it can be seen that the reaction rate of FFCA in Ru^+2.9^ is about 10 times that of Ru^+1.8^, which to some extent indicates the high yield of FDCA in Ru^+2.9^ catalyst. The reaction rates of different substrates of the same catalyst are HMF > DFF > FFCA in order. It was found that the reaction rate of FFCA is the lowest among all substrates, indicating that the oxidation of -CHO on FFCA to -COOH on FDCA is relatively difficult and is the determining step in the entire reaction process [31]. Meanwhile, the turnover frequency (TOF) values of four catalysts are also listed in Table S1, among which the TOF of Ru^+2.9^ catalyst is 2000 h^−1^, about 4–5 times that of Ru^+1.1^ and Ru^+1.8^, representing that the Ru^+2.9^ catalyst with a central valence state exhibits the best catalytic activity among the four valence state catalysts. The above analysis shows that these four Ru catalysts with different valence states exhibit significant differences in kinetics, and Ru^+2.9^ exhibits the best performance.

### 2.3. Optimization of Ru^+2.9^ Catalyst Reaction Parameters

The Ru^+2.9^ catalyst, which demonstrated the best performance among the four Ru catalysts, was selected for further optimization of reaction conditions. Initially, the influence of the electrolyte pH value during the electrocatalytic process was investigated. The oxidation activity of the Ru^+2.9^ catalyst was evaluated in 0.1 M K_2_SO_4_, 0.1 M H_2_SO_4_, and 0.1 M KOH electrolytes with and without 20 mM HMF, DFF, or FFCA using LSV (Figure S1). During the electrocatalytic reaction of HMF, there is a competition reaction of water. The oxidation potential of water is biased towards the back in the absence of any substrate. However, when the substrates HMF, DFF, and FFCA were added, the current density of the substrate oxidation peak was significantly higher than that of water oxidation, indicating that the oxidation of HMF, DFF, and FFCA is more favorable than water oxidation. Furthermore, differences in the oxidation current density of substrates were observed under varying pH conditions, following the following order: K_2_SO_4_ > H_2_SO_4_ > KOH. This suggests that the conversion of HMF and the selectivity of products are influenced, to some extent, by the pH of the electrolyte [31]. For a more detailed comparison, the oxidation potentials of reactions in electrolytes with different pH values were determined using LSV. The oxidation potentials were found to be 0.95 V in K_2_SO_4_ (60 °C), 0.95 V in H_2_SO_4_ (60 °C), and 0.3 V in KOH (room temperature). The lower oxidation potential under alkaline conditions indicates that OH^-^ can facilitate the initial oxidation of HMF.

**Figure 4:**
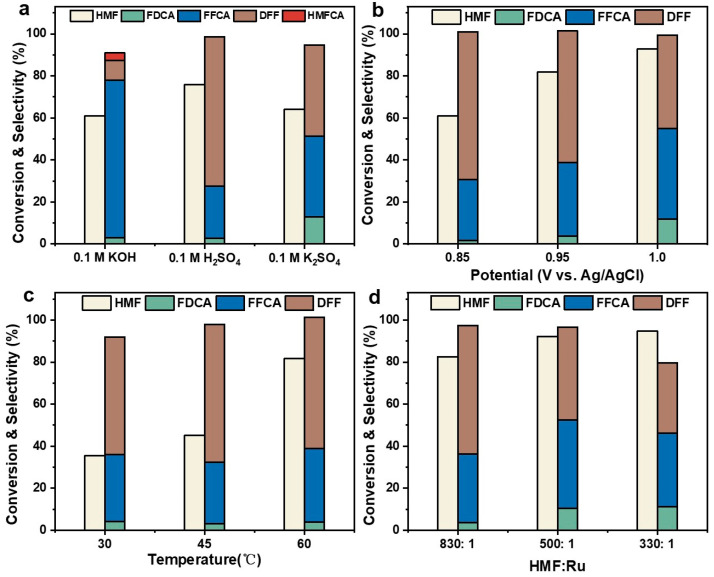
Influence of the (**a**) pH, (**b**) potential, (**c**) temperature, and (**d**) HMF/Ru molar ratio on the oxidation of HMF over the Ru^+2.9^ catalyst.

Constant potential testing experiments were conducted in various solutions according to the predetermined potentials mentioned above, and the obtained results are presented in Figure 4a. The intermediates produced during the electrooxidation of HMF in K_2_SO_4_ and H_2_SO_4_ solutions were exclusively DFF and FFCA, suggesting that the reaction pathways in these electrolytes are primarily mediated by DFF. In contrast, along with DFF and FFCA, a small amount of HMFCA was also detected in KOH electrolyte. This observation indicates that the electrochemical process in the alkaline electrolyte follows the two series paths of DFF and HMFCA dominated by DFF (HMF → DFF → FFCA → FDCA and HMF → HMFCA → FFCA → FDCA).

After 2 h of reaction, the majority of the products under alkaline conditions are concentrated in the intermediate compound FFCA, with the FDCA selectivity being minimal. However, the selectivity of DFF is higher in neutral K_2_SO_4_ and acidic H_2_SO_4_ solutions, indicating that HMF is more likely to oxidize -OH functional groups to form the intermediate product DFF in non-alkaline solutions. For long-term reactions, under the high conversion of HMF, FFCA selectivity was still observed to be as high as 89.1% in KOH solution, while FDCA selectivity was only 4.5% (Figure S2). It indicates that the addition of base will inhibit the conversion of FFCA to FDCA, thereby reducing the yield of FDCA. The conversion of FDCA in H_2_SO_4_ solution is slow and, even after 24 h, the yield of FDCA is 42.9%. There is also a small amount of DFF and a large amount of FFCA that has not been converted. H^+^ in the solution inhibits the generation of FDCA. Comparing the three solutions, it can be found that the final yield and activity of FDCA are highest in 0.1 M K_2_SO_4_.

To further understand the reaction pathway on the Ru^+2.9^ catalyst, HMF, DFF, and FFCA were used as reactants for kinetic experiments to study their initial reaction rates under different pH conditions. The conversion of reactants was controlled below 20%, and the final calculation results are shown in Table 1. The reaction rate of DFF in KOH is as high as 558 mol∙mol_Ru_^−1^∙h^−1^, which is about twice that of K_2_SO_4_ and 10 times that of H_2_SO_4_. This result is consistent with the high selectivity of FFCA in the alkaline product distribution above. On the other hand, it can be seen that the initial reaction rates of HMF and FFCA in K_2_SO_4_ are higher than those of the same substrate in H_2_SO_4_ and KOH. The initial conversion rate of HMF in K_2_SO_4_ is 388 mol∙mol_Ru_^−1^∙h^−1^, which is about 2.5 times that in H_2_SO_4_ and KOH. The initial conversion rate of FFCA in K_2_SO_4_ is 167 mol∙mol_Ru_^−1^∙h^−1^, which is about four times that in H_2_SO_4_ and KOH, indicating the high yield of FDCA in K_2_SO_4_. Based on the above data, it was found that the oxidation of -CHO to -COOH in FFCA is the slowest, and the process from FFCA to FDCA is the rate control step. Promoting the oxidation of -CHO in FFCA plays a crucial role in improving the efficiency of Ru-based catalysts. The above analysis shows that 0.1 M K_2_SO_4_ solution is more suitable for Ru^+2.9^ catalyst electrocatalytic oxidation of HMF to FDCA.

Potential is considered one of the important factors in the electrocatalytic oxidation of HMF to generate FDCA. The reaction activity of HMF electrocatalytic oxidation was compared between the oxidation potentials of 0.85 V and 1.0 V, as shown in Figure 4b. Within 2 h of the initial reaction, increasing the reaction voltage from 0.85 V to 1.0 V resulted in a corresponding increase in the conversion of HMF from 61% to 92.9%, while the selectivity of DFF decreased from 70.3% to 44.4%. At the same time, -OH on HMF is easily oxidized at lower reaction voltages. Increasing the voltage appropriately can promote the further conversion of aldehyde groups on DFF to carboxyl groups on FFCA and FDCA. This means that the oxidation of aldehyde groups requires applying higher reaction voltages to facilitate the generation of the target product FDCA.

Further extending the time to 24 h (Figure S3), the yield of FDCA was 90.2% and 54% at 0.95 V and 1.0 V, respectively. The increased voltage resulted in a decrease of about 40% in FDCA yield. We believe that the reason for this result is that excessive electrode potential during a long reaction process can lead to polymerization of reactants and intermediate products, forming by-products such as humus [5]. Based on the discussion of the above results, 0.95 V was ultimately determined as the appropriate reaction voltage.

Figure 4c shows the variation of HMF conversion and product distribution with temperature, and it is observed that temperature has a significant impact on HMF conversion and product distribution. The reaction temperature increased from 30 °C to 60 °C. After 2 h of reaction, the HMF conversion significantly increased from 35.5% to 92.3%. After 24 h of reaction, the HMF conversion remained at 100%, and the FDCA yield increased from 29.6% to 90.2%, as shown in Figure S4. At a temperature of 60 °C, the intermediate product only had a selectivity of 4.6% for FFCA. Temperature can effectively promote the conversion process of DFF and FFCA to FDCA, which has a significant impact on the activity of electrocatalytic oxidation of HMF. However, considering the temperature tolerance of the reaction solution and the reference electrode used, 60 °C was ultimately chosen as the optimal reaction temperature.

The effect of the molar ratio of HMF/Ru on the electrooxidation of HMF to FDCA is shown in Figure 4d. It can be observed that the conversion of HMF increases with the increase in Ru content at the beginning, indicating that increasing the amount of Ru can promote the conversion of HMF. When the time was extended to 24 h (Figure S5) for complete conversion of HMF, a molar ratio of HMF/Ru = 500 resulted in a 90.2% FDCA yield. The FDCA obtained at a molar ratio of HMF/Ru = 330 was only 79.4%, indicating that the activity of HMF/Ru = 500 was better than that of HMF/Ru = 330. From the perspective of experimental cost, this article ultimately chose a molar ratio of HMF/Ru = 500 as the appropriate molar ratio for this reaction.

### 2.4. The Catalytic Ability of Ru^+2.9^ Catalyst

**Figure 5:**
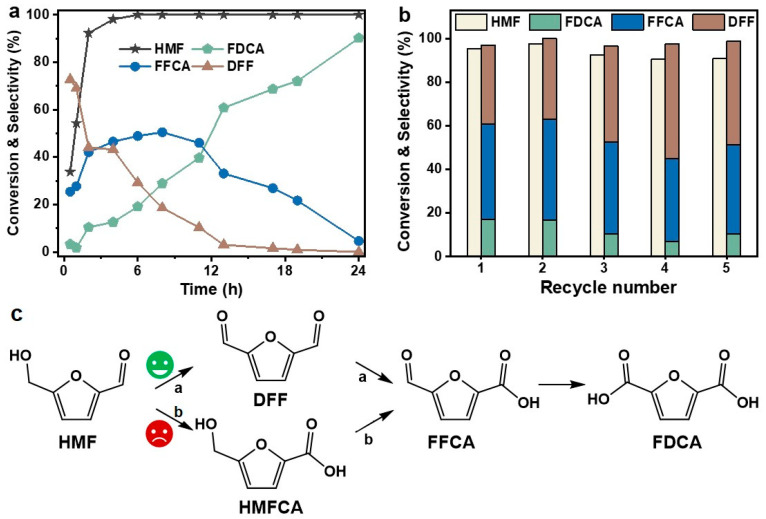
(**a**) The dynamic curve of HMF oxidation over the Ru^+2.9^ catalyst. (**b**) Recycling performance for Ru^+2.9^ catalyst. Reaction conditions: HMF/Ru: 500; HMF: 20 mM; electrolyte: 0.1 M K_2_SO_4_; temperature: 60 °C; potential: 0.95 V (vs. Ag/AgCl). (**c**) Schematic diagram of the reaction pathway for HMF oxidation.

Under optimized conditions, the dynamic curves between HMF and its oxidation intermediates and products were plotted, as shown in Figure 5a. Utilizing the Ru^+2.9^ catalyst, the HMF conversion approached 100% after 4 h. When the reaction was further extended to 24 h, the FDCA yield increased to a maximum of 90.2%. Notably, the final reaction solution exhibited a brown coloration. This indicates that the unstable HMF decomposed into dark brown humus by-products through cross-linking reactions under high temperature [32,33]. The resulting solution becomes acidic due to the formation of a large amount of FDCA.

The stability and product selectivity of the Ru^+2.9^ catalyst for the electrocatalytic oxidation of HMF in K_2_SO_4_ solution were investigated through cyclic reactions. As shown in Figure 5b, the reaction activity of the Ru^+2.9^ catalyst exhibited a slight decrease after five consecutive reaction cycles, demonstrating that the Ru^+2.9^ catalyst maintains relatively stable over repeated use. This finding highlights the Ru^+2.9^ catalyst’s potential as a durable material for electrocatalytic applications. Interestingly, only two intermediate products (DFF and FFCA), were detected via HPLC throughout the entire electrolysis process. This suggests that the electrocatalytic process likely follows the pathway HMF → DFF → FFCA → FDCA, as illustrated in Figure 5c.

### 2.5. Reaction Mechanism of HMF Electrooxidation

**Figure 6:**
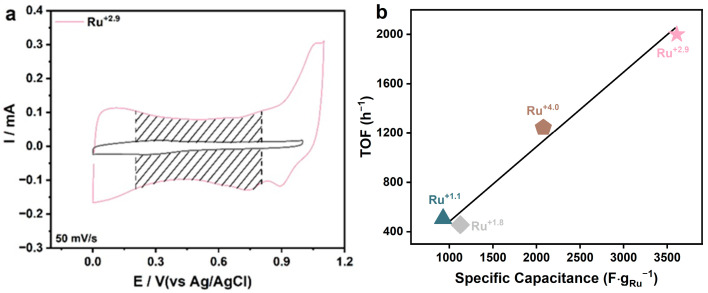
(**a**) Cyclic voltammograms of Ru^+2.9^ catalysts in 0.1 M K_2_SO_4_ with and without alcohol. (**b**) The relationship between specific capacitance of Ru and TOF of HMF over four Ru catalysts.

Catalysts with different surface oxidation states exhibit varying abilities to transport protons or electrons. Specific capacitance (SC) is a commonly used parameter for evaluating the proton (H^+^) and electron (e^−^) transfer abilities of catalysts, where higher values indicate more efficient proton and electron transfer on the catalyst surface [34]. Therefore, electrochemical methods were employed to measure the specific capacitance values of Ru catalysts with different valence states to evaluate their hydrogen transfer capabilities. Electrochemical cyclic voltammetry (CV) scanning was performed in a blank K_2_SO_4_ solution to evaluate the SC of the catalysts, and the results are shown in Figure 6 and Figure S6. The gray part under the CV curve represents the total charge stored during a complete scanning cycle. Notably, the specific capacitance of CNTs alone is significantly smaller compared to that of the loaded catalyst. Furthermore, there are significant differences in the specific capacitance of the four prepared Ru catalysts, with the Ru^+2.9^ catalyst displaying the highest SC. A high specific capacitance, such as that of the Ru^+2.9^ catalyst, indicates the presence of abundant active sites (e.g., Ru^3+^-OH) on its surface, which can accelerate β-hydrogen transfer and proton release during dehydrogenation reactions, while simultaneously facilitating rapid electron transfer to the electrode to sustain active site regeneration (Ru^3+^-H → Ru^3+^-OH). In contrast, low specific capacitance, such as that of pristine CNTs, results in weak charge storage capacity, leading to intermediate accumulation and reaction retardation. By optimizing the specific capacitance, the catalytic cycle efficiency can be enhanced, thereby increasing the generation rate and selectivity of target products, such as carboxylic acids.

By analyzing the correlation between the TOF of the reaction and the SC of the catalyst, a clear dependence between these parameters is observed. Among them, the Ru^+2.9^ catalyst exhibits the highest SC and TOF values, as shown in Figure 6b. This observation suggests a strong relationship between the catalytic performance of the catalyst and hydrogen transfer ability [35]. Specifically, the valence state of Ru in the catalyst profoundly influences the SC. A higher SC signifies a greater hydrogen transfer ability, which in turn results in enhanced catalytic activity. Among the four Ru catalysts with different valence states, the hydrogen transfer abilities follow the following order: Ru^+2.9^ > Ru^+4.0^ > Ru^+1.1^ ≥ Ru^+1.8^, a trend that aligns with their catalytic activity. The Ru^+2.9^ catalyst with a central valence state exhibits the highest activity. This finding is further supported by the calculations of Fumiya Nikaidou et al. [36], who determined that the Ru^+2.9^ active center indeed exhibits superior catalytic activity.

**Scheme 1:**
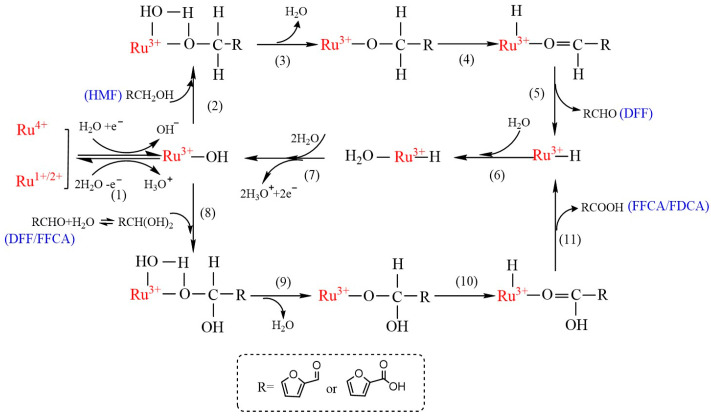
Proposed electrocatalytic cycles of the alcohol electrooxidation.

The reaction mechanism [37] for the electrocatalytic oxidation of HMF using Ru catalyst is proposed in Scheme 1: (1) Ru^n+^ dissociates water to form active center Ru^3+^-OH. Therefore, on Ru-based catalysts, trivalent Ru is more favorable for the progress of the reaction. In steps (2) and (3), the ligand exchange dehydration process between the active center Ru^3+^-OH and alcohols forms Ru^3+^-alkoxides. In steps (4) and (5), β-hydrogen is transferred from C atoms to Ru alcohol, followed by the breaking of Ru^3+^-O bonds to generate aldehydes and Ru^3+^-H compounds, along with the release of one H^+^ and one e^-^. In steps (6) and (7), Ru^3+^-H completes the recycling of active center Ru^3+^-OH with the help of water and the dehydration step further removes one H^+^ and one e^−^. (8) The aldehyde generated in process (5) is hydrolyzed to form a diol intermediate, and then a ligand exchange process occurs between a hydroxyl group on the diol intermediate and the active Ru^3+^-OH to form Ru^3+^-alkoxides, releasing 2H^+^ and 2e^−^ in two steps. The four removed electrons (e-) are transferred to the anode via the external circuit. Concurrently, the Ru^3+^ active centers are reduced yet rapidly regenerated through the oxidation of water, enabling the Ru^3^+-OH complex to cyclically participate in the reaction while the electrons are ultimately transferred out through the external circuit. Processes (9) (10), and (11) are the same as processes (3), (4), and (5), respectively.

In the electrocatalytic process of converting alcohols to carboxylic acids mentioned above, four protons on the substrate are removed, and the active sites are reduced by four electrons. Therefore, the proton and electron transfer ability (hydrogen transfer ability) of the catalyst is very important [35], which will affect the cyclic regeneration of the active sites of Ru^3+^-OH, that is, strong hydrogen transfer ability means better electrochemical activity.

## 3. Materials and Methods

### 3.1. Materials

RuCl_3_·xH_2_O (Ru content 35–42%, Aladdin, Shanghai, China), carbon nanotubes (CNTs, ShenzhenNano, Shenzhen, China), H_2_SO_4_ (95%, SCR), HNO_3_ (65%, SCR), KOH (95%, Aladdin), K_2_SO_4_ (Aladdin), H_2_O_2_ (30%, Macklin, Shanghai, China), NaBH_4_ (98%, Aladdin), PVP (40,000), Nafion (Alfa Aesar, Ward Hill, MA, USA). KMnO_4_ (99.5%), citric acid (97%, Aladdin), Mn(Ac)_2_∙4H_2_O (99%, Energy Chemical, Nanjing, China), HMF (98%, J & K, Kawasaki, Japan), 2,5-diformylfuran (DFF, >98%, Aladdin), hydroxymethyl-2-furancarboxylic acid (HMFCA, 97%, Ark Pharm, Huaian, China), 5-formyl-2-furancarboxylic acid (FFCA, 98%, Ark Pharm), and FDCA (>98%, Aladdin).

### 3.2. Catalyst Preparation

#### 3.2.1. CNT Purification [38]

First, 3 g of CNTs were dispersed into 300 mL HNO_3_ under ultrasound, refluxed at 140 °C for 2 h, then washed with ultrapure water to neutral, and dried under vacuum at 110 °C for 12 h.

#### 3.2.2. RuO_2_/CNT Catalyst Preparation

- Ru-CNT-H_2_ catalyst preparation [39]

First, 400 mg of CNTs were added to an aqueous solution of RuCl_3_·xH_2_O at room temperature, the solid was dried in air at 70 °C for 12 h to form the precursor. The precursor was then subjected to a temperature-programmed reduction treatment in a tubular furnace: heated to 300 °C at a rate of 5 °C/min and maintained under a 10 vol% H_2_/Ar mixed gas flow (50 mL/min) for 3 h to activate the metallic components. The catalyst was labeled as Ru-1.

- NaBH_4_ method [40,41]

RuCl_3_·xH_2_O precursor was dispersed in a polyvinylpyrrolidone mixed solution (MeOH: H_2_O = 1/1, in ml. PVP/Ru = 20/1, in mol) under stirring and then a freshly made NaBH_4_ solution (NaBH_4_/Ru = 1/1, in mol.) was added dropwise to obtain Ru nano-colloidal. The mixed solution was stirred for 6 h at room temperature and the CNTs were added to the mixed solution and stirred for 2 h. The catalyst was washed with hot water and dried under vacuum at 70 °C for 12 h. Finally, the catalyst was annealed in a tubular furnace under N_2_ flow (50 mL/min). The thermal treatment involved heating to 600 °C at a ramping rate of 5 °C/min and holding for 3 h. The catalyst was labeled as Ru-2.

- RuCl_3_-CNT catalyst preparation [42]

First, 400 mg of CNTs were added to an aqueous solution of RuCl_3_·xH_2_O at room temperature, the solid was dried under vacuum at 70 °C for 5 h, and the compressed samples were subjected to plasma-assisted reduction using a high-frequency alternating current (AC) power source (frequency: 10 Hz, power: 35 W) under an argon atmosphere (plasma-forming gas flow rate: 20 mL/min) for 30 min. The catalyst was labeled as Ru-3.

- RuO_2_-CNT catalyst preparation

First, 400 mg of CNTs were dispersed in RuCl_3_·xH_2_O under ultrasound for 2 h, then hydrogen peroxide was added at the rate of 12 mL/h using a microsyringe, heated at 80 °C for 4 h, washed in ultra-pure water and dried in a vacuum drying oven at 110 °C for 12 h, and finally the dried sample was ground into fine powder and calcined in a tubular furnace under N_2_ flow (50 mL/min). The thermal treatment involved heating to 600 °C at a ramp rate of 5 °C/min, followed by holding for 2 h. The catalyst was labeled as Ru-4.

#### 3.2.3. Catalyst Characterization

The morphology and particle size distribution of the catalyst were observed by HR-TEM with a JEOL JEM 2100F (Akishima, Japan). The specific operation method is as follows: a certain amount of sample is weighed and ultrasonically dispersed in acetone and then dripped on a microgrid copper net for drying. The loadings of metal species for all catalysts were measured via an ICP-OES (PerkinElmer Avio 200, Springfield, IL, USA). XRD patterns were obtained on the Bruker AXS D8 Advanced Focus diffractometer (Bremen, Germany). The diffraction data were gathered with a 0.02-degree (2θ) resolution at a current of 20 mA and accelerating voltage of 40 mV. XPS was conducted with a Thermo Scientific K-Alpha+ (Waltham, MA, USA) with monochromatic Al Kα radiation (hν = 1486.6 eV) under 100 eV pass energy and 1 eV energy step. Temperature-programmed reduction (TPR) analysis was performed on the AutoChem II 2920 (Micromeritics, Norcross, GA, USA). Here, a 50 mg sample was pretreated with Ar at 150 °C for 1 h, then analyzed in flowing 10% H_2_/Ar using a 5 °C/min ramp up to 300 °C and then cooled to 25 °C. Hydrogen chemisorption experiments were performed on the AutoChem II 2920 (Norcross, GA, USA). Here, 0.05 g of catalyst was placed in a quartz reactor before pulse chemical sorption, reduced at 550 °C for 3 h, and cooled to 50 °C at a rate of 10 °C/min in flowing 1% H_2_/Ar. Then, an H_2_ adsorption test was performed at this temperature and repeated at 2 min intervals until the signals on the detector became identical.

#### 3.2.4. Electrochemical Catalysis Oxidation Reaction

All electrochemical reactions are directed in a three-electrode system on the CHI660E electrochemical workstation (CH Instruments Inc., Shanghai, China). Pt foil and a Ag/AgCl electrode were used as the counter and reference electrodes, respectively, and the working electrode of CV experiments and LSV experiments is a glassy carbon electrode (20 µL 2 mg/mL catalyst dripping). The constant potential electrolysis test was conducted in an H-type electrolytic cell. Two semi-electrolytic cells were separated by a Nafion membrane, and the working electrode was carbon paper coated with catalyst. Here, 0.6 mmol HMF was added to the 30 mL K_2_SO_4_ aqueous solution and oxidized at a set temperature and voltage. The anode, cathode, and the overall reactions are summarized in the following equations:

Anode: HMF + 6OH^−^ → FDCA + 4H_2_O + 6e^–^ (1)

Cathode: 6H_2_O + 6e^−^ → 3H_2_ + 6OH^−^ (2)

Overall: HMF + 2H_2_O → FDCA + 3H_2_ (3)

#### 3.2.5. Analyses of Products

The oxidation products were analyzed by HPLC (Shimadzu LC-20A, Kyoto, Japan) with a chromatographic column (Shodex SUGARSH-1011, Tokyo, Japan), and the 20 µL electrolyte was diluted with ultra-pure water four times for analysis. The samples were placed in the autosampler (SIL-20A) with an injection volume of 5 μL. All collected samples are filtered through a PES filter (0.22 μm). HPLC parameters were set as: the mobile phase was 0.5 mmol/L H_2_SO_4_ with a flow rate of 0.9 mL/min, the sampling time was 35 min, and the column temperature is set at 40 °C.

The conversion, selectivity, yield, TOF, and Faraday efficiency (FE) were defined according to the following formulas, respectively:

(4) $$\mathrm{Conversion}\;(\%)=\frac{\mathrm{mol}\;\mathrm{of}\;\mathrm{HMF}\;\mathrm{converted}}{\mathrm{mol}\;\mathrm{of}\;\mathrm{HMF}\;\mathrm{initial}}\;\times \;100\%$$

(5) $$\mathrm{Selectivity}\;(\%)=\frac{\mathrm{mol}\;\mathrm{of}\;\mathrm{product}}{\mathrm{mol}\;\mathrm{of}\;\mathrm{HMF}\;\mathrm{converted}}\;\times \;100\%$$

(6) $$\mathrm{Yield}\;(\%)=\frac{\mathrm{mol}\;\mathrm{of}\;\mathrm{product}}{\mathrm{mol}\;\mathrm{of}\;\mathrm{HMF}\;\mathrm{initial}}\;\times \;100\%$$

(7) $$\mathrm{FE}\;(\%)=\frac{\mathrm{mol}\;\mathrm{of}\;\mathrm{FDCA}\;\mathrm{formed}}{\mathrm{total}\;\mathrm{charge}\;\mathrm{passed}/(F\;\times \;6)}\;\times \;100\%$$

where F is the Faraday constant (96,485 C∙mol^−1^).

## 4. Conclusions

In summary, a series of Ru-based catalysts with different valence states were prepared for the electrocatalytic oxidation of HMF to FDCA. Among the catalysts, the Ru^+2.9^ catalyst demonstrated superior performance, achieving a remarkable FDCA yield of 90.2% at 0.95 V (vs. Ag/AgCl). The high activity of Ru^+2.9^ was attributed to its optimal oxidation state, which enhanced hydrogen transfer and facilitated the regeneration of active sites. Mechanistic studies revealed that the reaction pathway proceeds via the intermediates DFF and FFCA, with the oxidation of FFCA to FDCA being the rate-determining step. Furthermore, the Ru^+2.9^ catalyst exhibited excellent stability and reusability over multiple cycles. This work not only provides a cost-effective and sustainable method for FDCA production but also offers valuable insights into the role of catalyst valence states in electrocatalytic biomass upgrading.

## Figures and Tables

**Table 1:** The initial reaction rates for the conversion of intermediates and HMF over Ru^+2.9^ in different electrolytes.

Electrolyte	Initial Reaction Rate (mol∙mol_Ru_^−1^∙h^−1^)			
	HMF	DFF	HMFCA	FFCA
K_2_SO_4_	388	296	/	167
H_2_SO_4_	161	52	/	40
KOH	156	558	117	49

## Data Availability

The raw data supporting the conclusions of this article will be made available by the authors on request.

## Supplementary Materials

The following supporting information can be downloaded at: https://www.mdpi.com/article/10.3390/molecules30081780/s1, Table S1: The result from characterization of the catalysts; Figure S1: LSVs of Ru^+2.9^ catalyst in (a) K_2_SO_4_, (b) H_2_SO_4_, (c) KOH at a scan rate of 2 mV/s; Figure S2: Influence of pH on the oxidation of HMF over the Ru^+2.9^ catalyst. Reaction conditions: HMF/Ru: 500; HMF: 20 mM; temperature: 60 °C; potential: 0.95 V (vs. Ag/AgCl); time: 24 h; Figure S3: Influence of potential on the oxidation of HMF over the Ru^+2.9^ catalyst. Reaction conditions: HMF/Ru: 500; HMF: 20 mM; temperature: 60 °C; electrolyte: 0.1 M K_2_SO_4_; time: 24 h; Figure S4: Influence of temperature on the oxidation of HMF over the Ru^+2.9^ catalyst. Reaction conditions: HMF/Ru: 500; HMF: 20 mM; potential: 0.95 V (vs. Ag/AgCl); electrolyte: 0.1 M K_2_SO_4_; time: 24 h; Figure S5: Influence of temperature on the oxidation of HMF over the Ru^+2.9^ catalyst. Reaction conditions: HMF: 20 mM; potential: 0.95 V (vs. Ag/AgCl); temperature: 60 °C; electrolyte: 0.1 M K_2_SO_4_; time: 24 h; Figure S6: Cyclic voltammograms of four Ru catalysts in 0.1 M K_2_SO_4_ with 50 mV/s.
