# Supplementary material for: Electrocatalytic Oxidation of HMF to FDCA over Multivalent Ruthenium in Neutral Electrolyte
Source: Molecules. 2025 Apr 15;30(8):1780. doi: 10.3390/molecules30081780 (PMC12029687; doi:10.3390/molecules30081780)
Supplement: Supplementary file 1 [file molecules-30-01780-s001.zip › molecules-3537861-supplementary.pdf]

Supporting Information

Linear Sweep Voltammetry and Cyclic voltammetry

The LSV and cyclic voltammetry in this experiment were performed using a three-electrode system, with saturated Ag/AgCl as the reference electrode, platinum plates as the counter electrode, and a glassy carbon electrode with catalyst droplets as the working electrode (preparation method: after cleaning the surface of the glassy carbon electrode, 20 μL 2 mg/mL of catalyst solution was dropped, and after the surface was dried, it can be used for measurement). Parameter settings: scanning speed of 50 mV/s, number of scanning circles of 20, and scanning range set as needed.

Turnover frequency (TOF) calculation

$$TOF_{Ru}\left(h^{-1}\right)=\frac{\text{mol of HMF converted}}{m \times \omega_t \times D_{Ru} \times h} \times 101.07$$

Where m is quality of catalyst (g). ω_t is contend of metals (%). h is reaction time (h). D_{Ru} was calculated based on the hydrogen adsorption amount measured by H_2-TPR.

Table S1 The result from characterization of the catalysts.

Catalyst	Wt. (%)	AOS	Capacitance ($F\cdot g^{-1}$)	Dispersion (%)	TOF (h^{-1})
Ru-1	0.72	+1.1	927.68	17.9	507
Ru-2	0.68	+1.8	1126.55	16.8	457
Ru-3	0.88	+2.9	3610.34	19.6	2000
Ru-4	0.74	+4.0	2078.42	18.4	1245

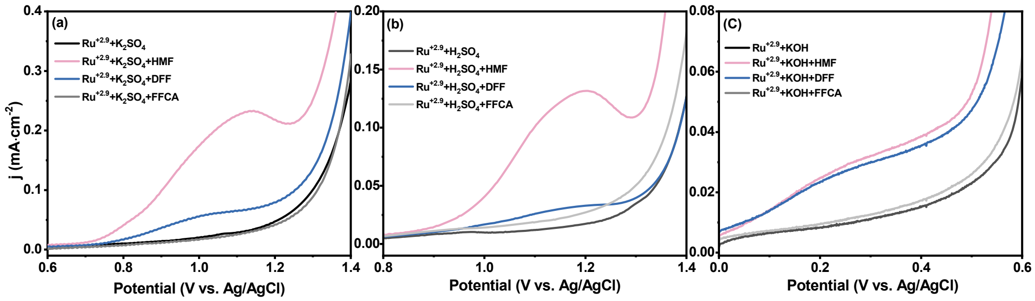

Figure S1. LSVs of $Ru^{+2.9}$ catalyst in (a) K_2SO_4 (b) H_2SO_4 (c) KOH at a scan rate of 2 mV/s.

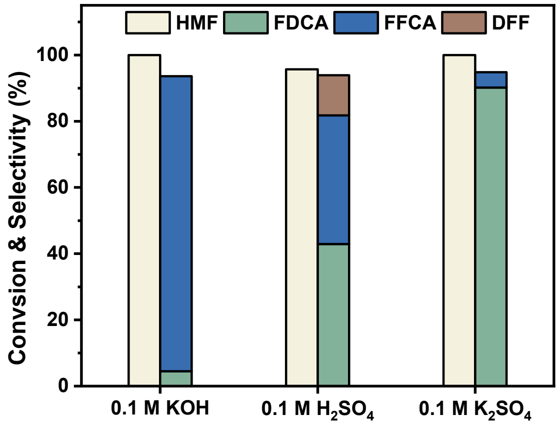

Figure S2. Influence of pH on the oxidation of HMF over the $Ru^{+2.9}$ catalyst. Reaction conditions: HMF/Ru: 500; HMF: 20 mM; temperature: 60 °C; potential: 0.95 V (vs. Ag/AgCl); time: 24 h.

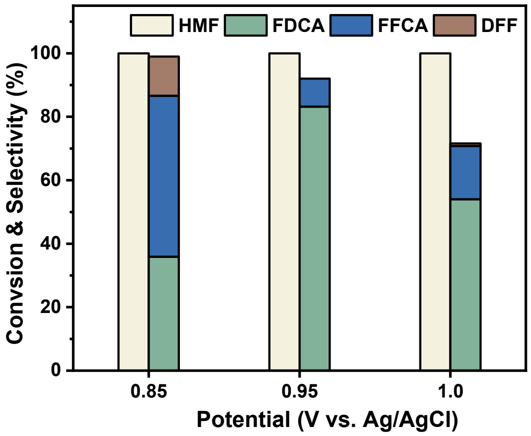

Figure S3. Influence of potential on the oxidation of HMF over the $Ru^{+2.9}$ catalyst. Reaction conditions: HMF/Ru: 500; HMF: 20 mM; temperature: 60 °C; electrolyte: 0.1 M K_2SO_4; time: 24 h.

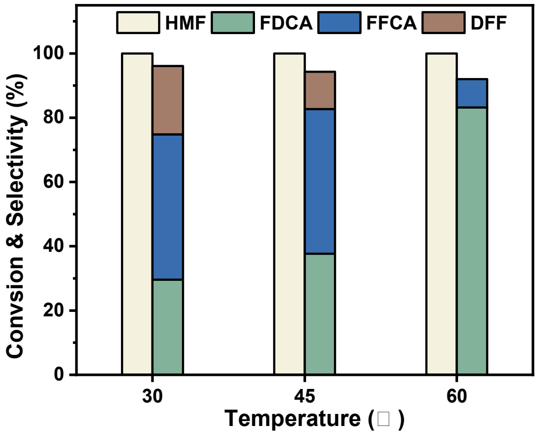

Figure S4. Influence of temperature on the oxidation of HMF over the $Ru^{+2.9}$ catalyst. Reaction conditions: HMF/Ru: 500; HMF: 20 mM; potential: 0.95 V (vs. Ag/AgCl); electrolyte: 0.1 M K_2SO_4; time: 24 h.

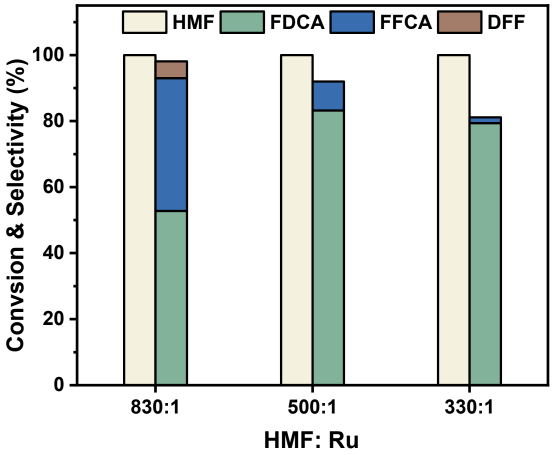

Figure S5. Influence of temperature on the oxidation of HMF over the $Ru^{+2.9}$ catalyst. Reaction conditions: HMF: 20 mM; temperature: 60 °C; potential: 0.95 V (vs. Ag/AgCl); electrolyte: 0.1 M K_2SO_4; time: 24 h.

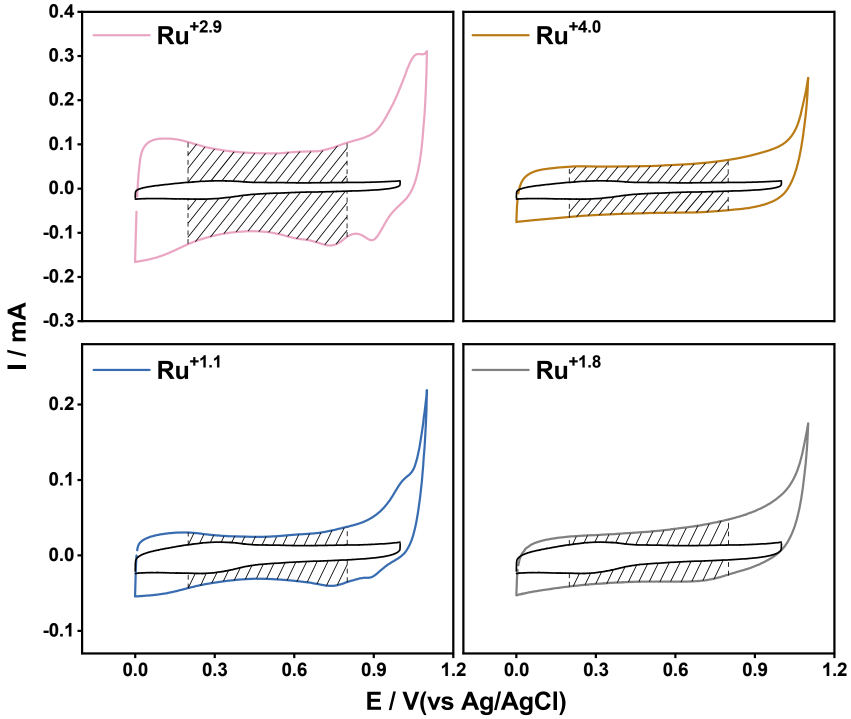

Figure S6 Cyclic voltammograms of four Ru catalysts in 0.1 M K_2SO_4 with 50 mV/s.
